# SMAC mimetics sensitize HIV-infected cells to oncolytic virus-mediated death

**DOI:** 10.3389/fimmu.2025.1665811

**Published:** 2025-12-18

**Authors:** Bengisu Molyer, Yasmeen Ameeriar, Jonathan B. Angel

**Affiliations:** 1 Inflammation and Chronic Disease Program, Ottawa Hospital Research Institute, Ottawa, ON, Canada; 2 Department of Biochemistry, Microbiology and Immunology, University of Ottawa, Ottawa, ON, Canada; 3 Department of Translational and Molecular Medicine, University of Ottawa, Ottawa, ON, Canada; 4 Leslie Dan Faculty of Pharmacy, University of Toronto, Toronto, ON, Canada; 5 Department of Medicine, Division of Infectious Diseases, The Ottawa Hospital, Ottawa, ON, Canada

**Keywords:** HIV, SMAC mimetics, oncolytic viruses, MG1, VSV

## Abstract

Elimination of latently and persistently HIV-infected cells is one of the main barriers to finding a cure for HIV. We have demonstrated that cells latently/persistently infected with HIV impair interferon signaling, which makes them susceptible to selective infection and killing by the oncolytic virus (OV) MG1. Sensitizing these cells to MG1-mediated killing can be expected to make MG1 a more effective therapeutic. As small-molecule second mitochondria-derived activator of caspases (SMAC) mimetics have been shown to increase OV-mediated death in cancer models, we used the SMAC mimetics LCL-161 and birinapant alongside MG1 to enhance the killing of HIV-infected cell lines and monocyte-derived macrophages (MDMs). We show that SMAC mimetics enhance MG1-mediated death, but that this is not a result of an increase in OV infection. This cell death occurs via both caspase-dependent and caspase-independent mechanisms and is not completely dependent on tumor necrosis factor alpha (TNFα). Together, these results show that the use of SMAC mimetics alongside OVs may be a viable strategy to eradicate latently/persistently HIV-infected cells.

## Introduction

One of the main challenges in human immunodeficiency virus-1 (HIV) cure research is the existence of long-lived latently infected CD4^+^ T cells and persistently infected macrophages. These cells are difficult to specifically target as markers that allow them to be distinguished from their healthy counterparts have yet to be identified. Our group has previously demonstrated that both latently HIV-infected CD4^+^ T cells and persistently HIV-infected monocyte-derived macrophages (MDMs) have defects in their interferon (IFN) signaling, which makes these cells permissive to killing by the IFN-sensitive oncolytic rhabdovirus MG1 (1, 2).

Sensitizing cells to killing by MG1 is an important step in the development of MG1 as a potential therapeutic. Studies in cancer have shown that oncolytic viruses (OVs), such as the rhabdovirus vesicular stomatitis virus (VSV) (3–5) or the alphavirus M1 (6), can be used alongside second mitochondria-derived activators of caspases (SMAC) mimetics to sensitize cancer cells to OV-mediated killing. SMAC mimetics are small pro-apoptotic molecules that mimic the N-terminal of the endogenous SMAC proteins in the cells. SMAC proteins bind to the inhibitors of apoptosis proteins cIAP1/2 and XIAP in the cell, resulting in their sequestration or degradation. This, in turn, enhances the caspase activity and ultimately results in cell death (7, 8). SMAC mimetics have been shown to selectively kill HIV-infected cells in a tumor necrosis factor alpha (TNFα)-dependent (9–11) or TNFα-independent manner (12) and to reverse the HIV latency (13, 14) in different models.

As HIV infection dysregulates the apoptotic responses in infected cells, in part by upregulating the inhibitors of apoptosis proteins (12, 15–18), and that SMAC mimetics can synergize with OVs to kill cancer cells, we hypothesized that OVs and SMAC mimetics may synergize to kill HIV-infected cells. Here, we show that SMAC mimetics can sensitize HIV-infected cell lines and *in vitro* HIV-infected MDMs to enhance MG1-mediated cell death.

## Materials and methods

### Media

Gibco^®^ Roswell Park Memorial Institute 1640 medium (RPMI 1640) with phenol red indicator, Gibco^®^ Dulbecco’s modified Eagle’s medium (DMEM), and OPTI-MEM reduced serum medium were purchased from Thermo Fisher Scientific (Waltham, MA, USA). Endotoxin-free Dulbecco’s phosphate-buffered saline (PBS), pH 7.4, was purchased from Millipore-Sigma (Burlington, MA, USA). The reagents used to supplement the media were heat-inactivated fetal bovine serum (FBS), penicillin (100 U/ml) and streptomycin (10 μg/ml) (PenStrep), l-glutamine (Thermo Fisher Scientific), and heat-inactivated human AB serum (Valley Biomedical, Winchester, VA, USA).

### Chemicals

Recombinant human M-CSF (carrier-free) was purchased from BioLegend (cat. no. 574802; San Diego, CA, USA). Human TNFα recombinant protein (cat, no. PHC3015) was purchased from Thermo Fisher Scientific. The bivalent SMAC mimetic AEG-40730 (cat. no. 5330) was purchased from Tocris Bioscience (Bristol, UK). LCL-161 (cat. no. S7009) and birinapant (cat. no. S7015) were purchased from Selleck Chemicals (Burlington, ON, Canada). The pan-caspase inhibitor Z-VAD-FMK (cat. no. 2163) was purchased from Bio-Techne (Minneapolis, MN, USA). Necrostatin-1s (cat. no. 17802) was purchased from Cell Signaling Technology (Danvers, MA, USA).

### Cell culture

J1.1 cells (ARP-1340) were obtained through the National Institutes of Health (NIH) HIV Reagent Program, Division of AIDS, National Institute of Allergy and Infectious Diseases (NIAID), NIH, contributed by Dr. Thomas Folks. OM10.1 (ARP-1319) cells were obtained through the NIH HIV Reagent Program, Division of AIDS, NIAID, NIH, contributed by Dr. Salvatore Butera. Both cell lines were cultured in RPMI 1640 medium supplemented with 10% FBS, PenStrep, and l-glutamine (2 mM; R10 medium). The 293T cells (CRL-3216) were obtained from ATCC (Manassas, VA, USA) and cultured in DMEM supplemented with 10% FBS, PenStrep, and l-glutamine (2 mM). The cells were maintained at 0.2 × 10^5^–1 × 10^6^ cells/ml and passaged every 2–3 days. The MDMs were cultured in RPMI 1640 medium supplemented with 10% human AB serum and PenStrep (MDM medium).

### Generation of macrophages

Experiments requiring healthy volunteers were approved by the Ottawa Health Science Network Research Ethics Board (protocol no. 2005388-01H), and all participants provided written informed consent. All methods were performed in accordance with relevant guidelines and regulations. The macrophages were generated from healthy donor blood as described previously (2). Briefly, fresh blood was collected from healthy donors, and peripheral blood mononuclear cells (PBMCs) were isolated via density gradient isolation. Following this, the PBMCs were resuspended at 6.25 × 10^6^ cells/ml, plated in 100-cm^2^ untreated polystyrene tissue culture dishes (Sarstedt, Nümbrecht, Germany), and left to adhere for 2 h at 37°C. After the incubation, the plates were washed three times with endotoxin-free PBS and 10 ml of the RPMI 1640 medium supplemented with 10% human AB serum and PenStrep (MDM medium), and M-CSF (25 U/ml) was added to the plates. On day 3, the plates were washed with endotoxin-free PBS and 10 ml of the MDM medium was added. On day 8, the adherent MDMs were washed with endotoxin-free PBS, treated with 5 ml of Accutase (Millipore-Sigma) for 30 min at 37°C, and the cells gently pipetted off. The MDMs were then plated at 2 × 10^5^ cells/well in 24-well untreated polystyrene plates for further experiments.

### Virus amplification

The HIV NL4.3 BAL-IRES-HSA plasmid encoding the CCR5-tropic (R5-tropic) virus, which was obtained from Dr. Michel J. Tremblay at Université Laval, was amplified on 293T cells as described previously (2). In brief, 293T cells were transfected with the HIV NL4.3 BAL-IRES-HSA plasmid. Mock-infection stocks were prepared in parallel. At 48 h post-incubation, the supernatant was collected, centrifuged, and filtered sequentially via 0.45- and 0.22-μm polyvinylidene fluoride (PVDF) syringe filters (UltiDent Scientific, St. Laurent, QC, Canada). Infection with this reporter virus results in the presentation of mouse heat-stable antigen (HSA) on the cell surface, allowing for the differentiation between HIV-infected (HSA+) or bystander cells (HSA−). The MG1-eGFP recombinant OV was obtained from Dr. David Stojdl, and VSVΔ51 was obtained from Dr. Jean-Simon Diallo. Both were propagated on Vero cells as described previously (1). Briefly, Vero cells were grown to confluency and then infected with either OV at a multiplicity of infection (MOI) of 0.01. At 24 h post-infection (hpi), the supernatant was collected, centrifuged, and filtered through a 0.2-μm pore Nalgene bottle-top filter (Nalgene Nunc, Rochester, NY, USA). The filtered supernatants were centrifuged again in a high-speed centrifuge and the viral pellet resuspended in PBS.

### SMAC mimetic treatment

J1.1 and OM10.1 cells were plated at 1 × 10^6^ cells/ml in 500 µl in 24-well plates. The cells were then treated with different doses of the SMAC mimetic AEG-40730 (2–6 µM; Tocris Bioscience), LCL-161 (2–20 µM; Selleck Chemicals), or birinapant (1–2 µM; Selleck Chemicals) at a total volume of 1 ml in R10 medium. The cell viability and surface receptor expression were measured 48 h post-treatment by flow cytometry. The cell-free supernatants were collected to assess the p24 antigen concentration and the cytokine production.

HIV-infected MDMs were plated at 2 × 10^5^ cells/ml in untreated 24-well plates and then treated with the SMAC mimetic LCL-161 or birinapant at doses of 2–4 µM at a total volume of 1 ml in MDM medium. The frequency of HSA-expressing cells, the cell surface marker expression, and the cell viability were determined by flow cytometry.

### HIV infection of monocyte-derived macrophages

On day 9 of generation, the MDMs were washed once with prewarmed endotoxin-free PBS. HIV NL4.3 BAL-IRES-HSA was added to the cells at a ratio of 100 ng of p24 per 1 × 10^6^ MDM in 500 µl. After an overnight incubation at 37°C with 5% CO_2_, the volume of the cell medium was doubled to 1 ml using the MDM medium. The cells were then incubated for 6 days at 37°C with 5% CO_2_, with a 1/2 medium change performed at 3 days post-infection (dpi). At 6 dpi, the cells were prepared for experiments as outlined below.

### OV infection

J1.1 and OM1.1 cells were plated at 1 × 10^6^ cells/ml in 200 μl in 24-well plates and infected with MG1 at an MOI of 1 × 10^−4^ or VSV at an MOI of 1 in 300 μl R10 medium for 2 h, after which the medium was topped up to 1 ml/well. The cell viability was assessed by propidium iodide (PI) staining using flow cytometry.

HIV-infected MDMs were infected with MOI = 1 of MG1 or VSV in 250 µl for 2 h, after which the medium was topped up to 1 ml/well with the MDM medium. The frequency of HSA-expressing cells and the cell viability were determined by flow cytometry.

### Combination treatment with MG1 and SMAC mimetics

For concurrent treatment with MG1 and the SMAC mimetics, Jurkat cells, J1.1 cells, and OM10.1 cells were plated at 1 × 10^6^ cells/ml and were infected with MG1 (MOI = 1 × 10^−1^–1 × 10^−4^) for 2 h in 300 µl R10 medium. Following infection, the cells were topped up to 1 ml/well with an appropriate concentration of each SMAC mimetic (AEG-40730, LCL-161, or birinapant at a concentration range of 1–20 µM). At 48 hpi, the cell death, infection, and caspase-3/7 activation were measured by flow cytometry.

For MG1 infection followed by the SMAC mimetics, J1.1 and OM10.1 cells were infected with MG1 as described above for 24 h, and then the appropriate SMAC mimetic was added into the medium. The cell viability was assessed via flow cytometry at 48 hpi.

For the SMAC mimetic treatment followed by MG1 infection, J1.1 and OM10.1 cells were treated with the appropriate SMAC mimetic as described above for 24 h. Following this, the cells were collected and the supernatant containing the SMAC mimetic was saved for the next steps. The cells were resuspended in fresh medium and were infected with MG1 at 300 µl for 2 h, as described above. The cells were topped up to 1 ml with the appropriate SMAC mimetic containing-supernatant. At 24 hpi, the cell death, caspase-3/7 and caspase-1 activation, and infection were measured by flow cytometry.

HIV-infected MDMs were infected with MG1 at MOI 1 in 250 µl for 2 h in the MDM medium. Following this, the cells were topped up to 1 ml/well with an appropriate concentration of each SMAC mimetic (LCL-161 or birinapant at concentrations of 2–4 µM). At 48 hpi, the HSA frequency and cell death were measured via flow cytometry.

### Concurrent treatment with TNFα and SMAC mimetics

HIV-infected MDMs were plated at 2 × 10^5^ cells/ml and were treated with 10 ng TNFα for 2 h in 250 µl MDM medium to mimic viral infection conditions. Following this, the cells were topped up to 1 ml/well with 1 µM birinapant. At 48 h post-treatment, cell death was measured by flow cytometry.

### MG1 titration by standard plaque assay following concurrent treatment with birinapant

For viral titering of MG1 from the MG1- and birinapant-treated cells, J1.1 cells were infected with MG1 and concurrently treated with birinapant, as detailed above. At 48 hpi, the supernatants were collected and centrifuged to eliminate dead cells. Following this, Vero cells were infected with the supernatant at 10-fold dilutions. At 48 hpi, the plaques were visualized with crystal violet staining.

### Cytokine analysis

TNFα from the OM10.1 supernatants was measured using the Meso Scale Discovery U-PLEX platform (cat. no. K15067M-1). TNFα from the MDM supernatants was quantified using the Human TNF-α ELISA Kit (cat. no. KHC3011; Thermo Fisher Scientific). IFNα from the HIV-infected MDM supernatants was measured using the VeriKine Human IFN-Alpha ELISA Kit (cat. no. 411100-1) from Cedarlane (Piscataway, NJ, USA) following the manufacturer’s protocols.

### Flow cytometry

All flow cytometric analyses were performed using the Beckman Coulter CytoFLEX flow cytometer (Beckman Coulter, Brea, CA, USA) and analyzed on FlowJo v10.9.0 (BD Biosciences, Franklin Lakes, NJ, USA).

Prior to staining, the MDMs were detached using Accutase (60 min incubation at 37°C) and gentle pipetting. For the assessment of surface and intracellular markers, the detached MDMs were washed with PBS 1% BSA (460 × *g* for 5 min) and suspended in cold PBS plus 10% human AB serum and 20% normal goat serum (NGS) (Life Technologies, Carlsbad, CA, USA) and then incubated at 4°C for 20 min for blocking. The cells were then washed with PBS for further staining.

HSA staining was performed using either APC anti-mouse CD24 (HSA; clone 30-F1 cat. no. 113 CHF) or PE anti-mouse CD24 (HSA; clone 30-F1, cat. no. 138503) (both from BioLegend) and incubation at 4°C for 15 min (2 µl per tube for HSA-APC and 3 µl per tube for HSA-PE).

The expression of the low-density lipoprotein receptor (LDL-R) was measured using the human LDL-R PE-conjugated antibody (cat. no. FAB2148P; R&D Systems, Minneapolis, MN, USA), and programmed death-ligand 1 (PD-L1) was measured with the human CD274 (PD-L1) APC-conjugated antibody (cat. no. 393610; BioLegend).

The expression of annexin V was measured by staining with PE Annexin V (cat. no. 640908; Biolegend) in Annexin V Binding Buffer (cat. no. 422201; BioLegend) for 15 min at room temperature (0.5 μl/tube). The cell viability was measured by staining with a PI solution (0.1 μl/tube) (cat. no. 421301; BioLegend).

The activity of caspase-3/7 and caspase-1 was determined using the Far-Red Fluorescent FLICA^®^ 660 Caspase-3/7 (DEVD) Assay Kit (cat. no. 9125) and the Far-Red Fluorescent FLICA^®^ 660 Caspase-1 (YVAD) Assay Kit (cat. no. 9122; both from ImmunoChemistry Technologies, Davis, CA, USA) according to the manufacturer’s protocol. The infection percentage was measured based on the green fluorescent protein (GFP) expression. All cells were fixed in 1% paraformaldehyde (PFA) for 20 min at room temperature prior to analysis.

### HIV DNA PCR

A two-step nested PCR targeting CD3 and integrated HIV DNA was performed with previously described primer–probe sets (2, 19) using the CFX Connect™ Real-Time PCR Detection System (BioRad, Hercules, CA, USA). A standard curve was constructed by running the ACH-2 lysates in parallel with the samples to quantify the proviral HIV DNA copy number.

### RNA isolation and RNA-seq analysis

J1.1 cells were either left untreated, infected with MG1 at MOI = 1 × 10^−3^, treated with 1 µM birinapant, or concurrently treated with MG1 and birinapant for 48 h. RNA was extracted using the Qiagen RNeasy Mini Kit (cat. no. 74104).

RNA sample quality assessment was performed with the Fragment Analyzer (Agilent, Santa Clara, CA, USA) and the concentration measured with Qubit 3.0 (Thermo). Next-generation sequencing libraries were prepared with the Stranded mRNA Library Prep Kit (Illumina, San Diego, CA, USA) using 500 ng of the RNA input. Sequencing was performed with the NextSeq 2000 P3–100 Cycle Flow Cell (Illumina). The RNA sequencing (RNA-seq) dataset can be found at Gene Expression Omnibus (GEO) under GSE278469.

Analysis was performed using GENCODE v46 (20) annotations and the GRCh38 genome assembly. The nf-core/rnaseq version 3.14.0 (21) was used to generate the quality assessment and read the pseudocount table. Fold change analysis was performed using the R/Bioconductor library DESeq2 (22), with fold change shrinkage performed using the apeglm library (23). Enrichment analysis on the above generated fold change tables was performed using g:Profiler with the Gene Ontology (GO) biological term database, with term sizes between 2 and 500. The code used to generate the heatmaps can be found at https://bit.ly/4fQQARE.

### Bliss synergy score calculation

The Bliss synergy scores for J1.1 cells treated with MG1 and AEG-40730 or MG1 and LCL-161 were calculated using SynergyFinder 3.0 (24).

## Results

### SMAC mimetics sensitize latently HIV-infected cell lines to MG1-mediated death

**Figure 1:**
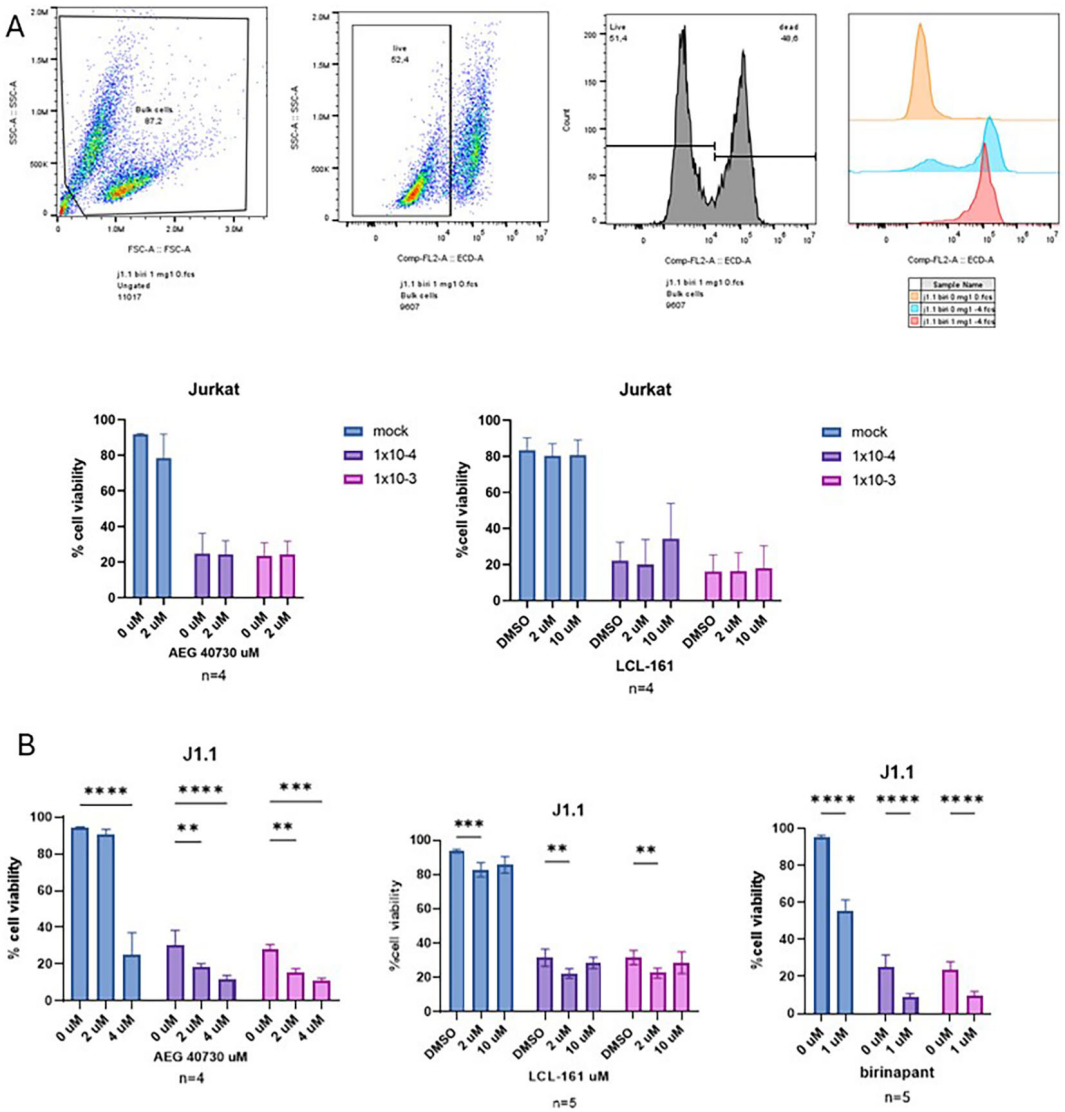
Combination treatment with MG1 and second mitochondria-derived activator of caspases (SMAC) mimetics kill HIV-infected cells. **(A)** Jurkat cells infected with MG1 at a multiplicity of infection (MOI) of 1 × 10^−3^ or 1 × 10^−4^ and treated with the SMAC mimetic AEG-40730 (2 µM) or LCL-161 (2 and 10 µM). Cell death was measured by flow cytometry via propidium iodide (PI) staining at 48 h post-infection (hpi). PI-negative cells were designated as “*viable*.” **(B)** J1.1 cells infected with MG1 at an MOI of 1 × 10^−3^ or 1 × 10^−4^ and treated with the SMAC mimetic AEG-40730 (2 and 4 µM), LCL-161 (2 and 10 µM), or birinapant (1 µM). Cell death was measured by flow cytometry via PI staining at 48 hpi. PI-negative cells were designated as “*viable*.” Data were analyzed using two-way analysis of variance with the Dunnett *post-hoc* test for multiple comparisons between the different SMAC mimetic doses in the same MG1 infection group. Data represent the mean value ± standard deviation of the mean. *n* values represent separate biological replicates. *p<.05 **p<.01, ***p<.001, ****p<.0001.

We have previously demonstrated that MG1 can selectively kill latently HIV-infected promonocytic U1 and promyelocytic OM10.1 cell lines, as well as persistently HIV-infected MDMs (1, 2). Here, the lymphocytic T-cell line Jurkat and its latently HIV-infected counterpart J1.1 cells were concurrently treated with the bivalent SMAC mimetic AEG-40730 or the monovalent SMAC mimetic LCL-161 and the oncolytic rhabdovirus MG1. Although Jurkat cells were more permissive to OV-mediated death compared with J1.1 cells, the addition of SMAC mimetics to MG1 infection significantly increased cell death in J1.1 cells, whereas no such increase was observed in the uninfected parental Jurkat cells (**Figure 1A**). Calculation of the Bliss synergy scores for J1.1 cells showed that MG1 and SMAC mimetics synergize at the lower dose of MG1 and the higher dose of SMAC mimetics (**Supplementary Figure S1**). Following these results, another bivalent SMAC mimetic, i.e., birinapant, was used alongside MG1 on J1.1 cells. Consistent with the above results, a significant increase in cell death was observed with the presence of this SMAC mimetic (**Figure 1B**). Subsequently, the SMAC mimetics were used alongside MG1 in the promyelocytic latently HIV-infected cell line OM10.1. With this cell line, which is less susceptible to MG1-mediated killing (25), the SMAC mimetics did not enhance cell death (**Supplementary Figure S2**).

As previous studies in cancer have shown that the order of administration of SMAC mimetics and MG1 may affect the cell viability outcomes (4, 5), we evaluated whether pretreatment (rather than concurrent treatment) with SMAC mimetics would increase MG1-mediated cell death. Similarly to when the cells were concurrently treated with the SMAC mimetics and MG1, we observed an increase in cell death in J1.1 cells that were pretreated with AEG-40730 and LCL-161 prior to infection with MG1. Interestingly, in OM10.1 cells, an increase in cell death could only be seen when the cells were pretreated with AEG-40730 prior to MG1 infection (**Supplementary Figures S2, S3**), confirming that the order of administration of the SMAC mimetics and MG1 influences the cell killing *in vitro* and may have potential to eventually be used as a therapeutic.

### Cell death following MG1 and SMAC mimetic treatment is not blocked by inhibitors of apoptosis or necroptosis

**Figure 2:**
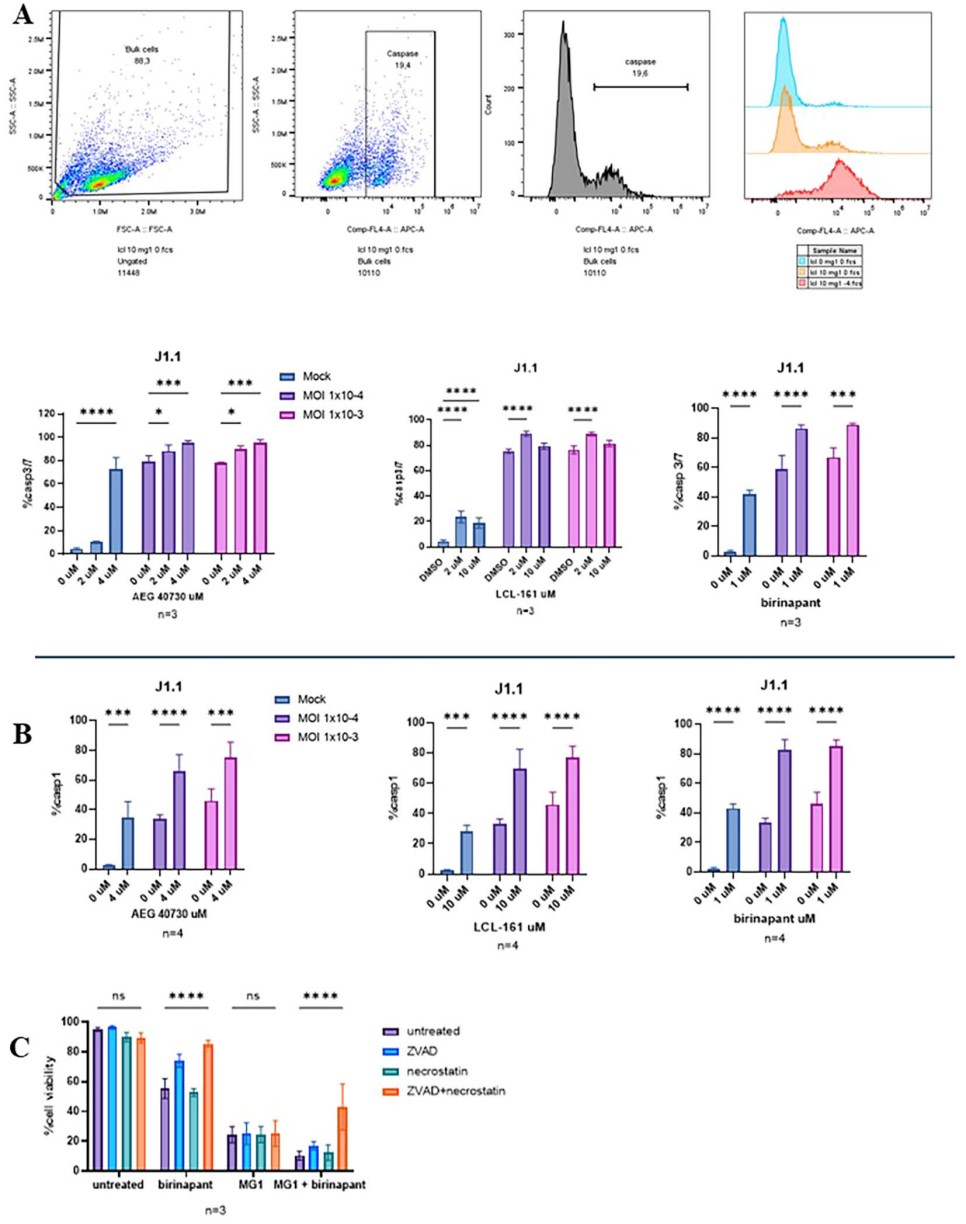
Although caspase-3/7 and caspase-1 were activated following treatment with MG1 and second mitochondria-derived activator of caspases (SMAC) mimetics, cell death could not be blocked by the apoptosis or necroptosis inhibitors. **(A, B)** J1.1 cells infected with MG1 at a multiplicity of infection (MOI) of 1 × 10^−3^ or 1 × 10^−4^ and treated with the SMAC mimetics AEG-40730 (2 and 4 µM), LCL-161 (2 and 10 µM), and birinapant (1 µM). Caspase-3/7 and caspase-1 activity was measured with FLICA 660 staining via flow cytometry. **(C)** J1.1 cells left untreated or pretreated with 50 µM Z-VAD-FMK, 50 µM necrostatin-1s, or with both 1 h prior to MG1 infection, birinapant (1 µM) treatment, or a combination of both. Cell death was measured by flow cytometry via propidium iodide (PI) staining. Data were analyzed using two-way analysis of variance with the Dunnett *post-hoc* test for multiple comparisons between the different SMAC mimetic doses in the same MG1 infection group. Data represent the mean value ± standard deviation of the mean. *n* values represent separate biological replicates. *p<.05, ***p<.001, ****p<.0001. ns, not significant.

Previously, it has been shown that SMAC mimetics kill latently HIV-infected CD4^+^ T cells via a combination of apoptosis and autophagy (12). Hence, we wanted to evaluate whether there is activation of the caspase-dependent cell mechanisms in cells treated with the SMAC mimetics and MG1. Cells concurrently treated with the SMAC mimetic AEG-40730, LCL-161, or birinapant and MG1 were stained with FLICA caspase-3/7 or FLICA caspase-1 at 48 hpi (**Figure 2A**). Interestingly, an increased caspase-3/7 and caspase-1 activity was observed with increased cell death, confirming that the caspase-dependent cell death mechanisms are activated following treatment with the SMAC mimetics and MG1 (**Figures 2A, B**).

RIPK1 has previously been shown to play a key role in the survival of HIV-infected macrophages (11). Thus, we wanted to evaluate whether inhibition of RIPK1 with necrostatin-1s and caspases with the pan-caspase inhibitor Z-VAD-FMK would decrease the SMAC mimetic- and MG1-induced cell death. J1.1 cells were treated with Z-VAD-FMK, necrostatin-1s, or a combination of both for 1 h prior to SMAC mimetic treatment only, MG1 infection only, or concurrent treatment with a SMAC mimetic and MG1. Cell death was measured at 48-hpi with PI staining. Pretreatment with Z-VAD-FMK alone slightly increased the cell viability in cells treated with birinapant, whereas pretreatment with Z-VAD-FMK and necrostatin-1s returned the cell viability levels close to that of the untreated cells. Neither Z-VAD-FMK nor necrostatin-1s pretreatment decreased cell death in MG1-infected cells. However, in cells treated with birinapant and MG1, pretreatment with Z-VAD-FMK and necrostatin-1s resulted in a significant decrease in cell death. This is most likely due to the cell death inhibitors blocking the SMAC mimetic-mediated cell death as the pretreatment did significantly increase the cell viability in cells only treated with birinapant, but did not increase the viability in cells only infected with MG1 (**Figure 2C**). Collectively, these results show that, although caspases become activated following treatment with the SMAC mimetics and MG1, cell death cannot be completely blocked by the pan-caspase inhibitor Z-VAD-FMK and necrostatin-1s, indicating that pathways other than apoptosis and necroptosis may play a role in cell death resulting from SMAC mimetic and MG1 treatment.

### SMAC mimetic treatment does not increase MG1 infection

**Figure 3:**
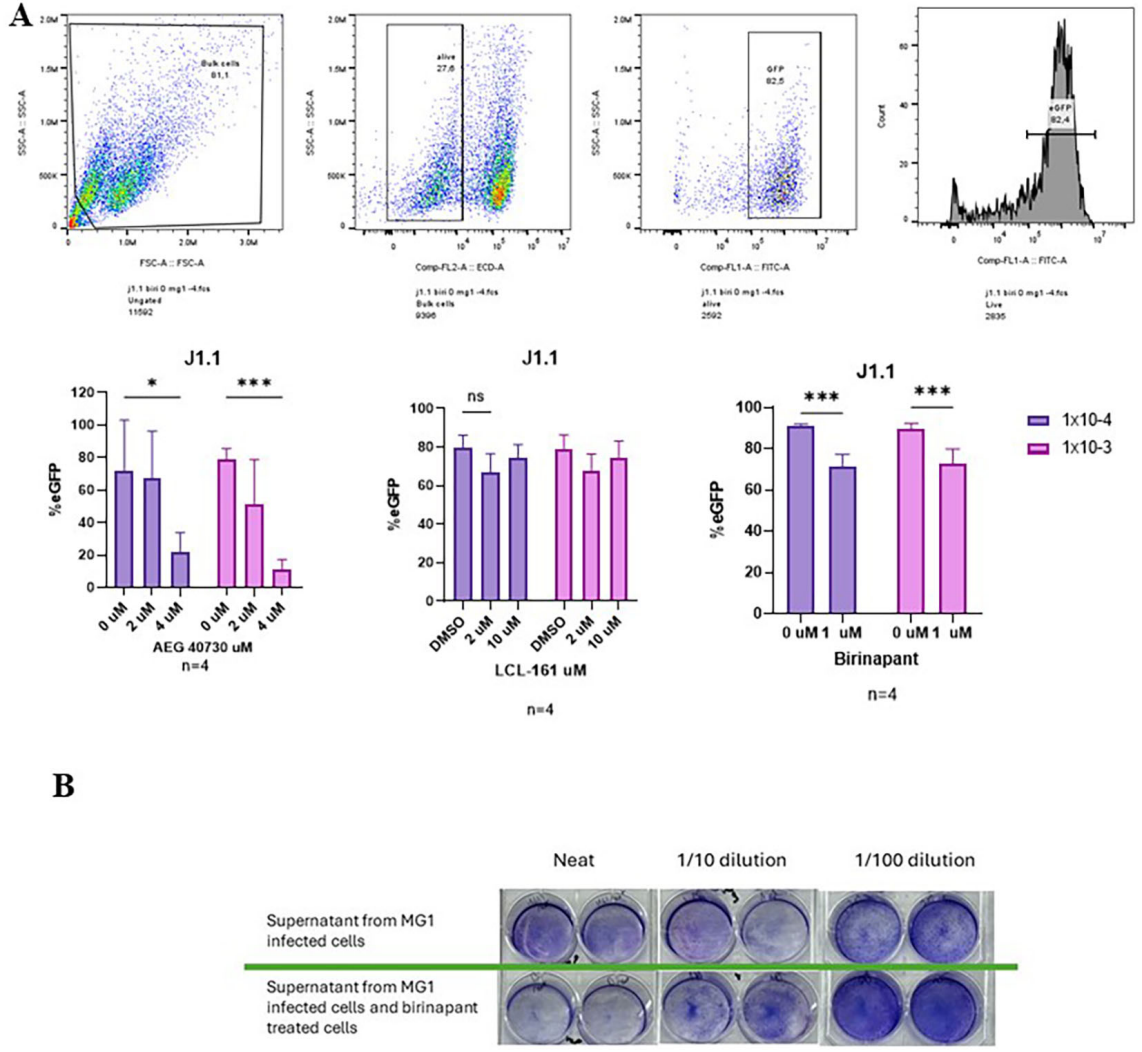
Infection percentage of J1.1 cells and plaque assay following treatment with MG1 and second mitochondria-derived activator of caspases (SMAC) mimetics. **(A)** J1.1 cells infected with MG1 at a multiplicity of infection (MOI) range 1 × 10^−3^–1 × 10^−4^ and concurrently treated with the SMAC mimetics AEG-40730 (2 and 4 µM), LCL-161 (2 and 10 µM), and birinapant (1 µM). Infection was measured at 48 h post-infection (hpi) by flow cytometry via green fluorescent protein (GFP) expression. Data were analyzed using two-way analysis of variance with the Dunnett *post-hoc* test for multiple comparisons between the different SMAC mimetic doses. Data represent the mean value ± standard deviation of the mean. *n* values represent separate biological replicates. **(B)** Supernatants from the MG1-infected or MG1-infected and concurrently birinapant-treated J1.1 cells collected at 48 hpi. The supernatants were used to perform plaque assays using Vero cells. 48hpi of Vero cells under agarose overlay, the plaques were fixed and stained.. Experiments performed, *n* = 3. Picture on *top* is a representative image. Data were analyzed using two-way analysis of variance with the Dunnett *post-hoc* test for multiple comparisons between the different SMAC mimetic doses in the same MG1 infection group. Data represent the mean value ± standard deviation of the mean. *n* values represent separate biological replicates. *p<.05, ***p<.001. ns, not significant

As we have shown that SMAC mimetics sensitize HIV-infected cells to MG1-mediated death, we investigated whether an increase in MG1 infection is the mechanism responsible for such. Thus, the GFP expression following 48 h of treatment with the SMAC mimetics and MG1 infection was determined. The expression of GFP appeared to decrease with the addition of SMAC mimetics (**Figure 3A**). As the decrease in GFP may be an unintended result of the high amount of cell death observed following combination treatment with MG1 and SMAC mimetics, we wanted to evaluate the MG1 virus production following treatment of J1.1 cells with SMAC mimetics. After 48 h of infection with MG1, with or without birinapant, the supernatants were collected and a plaque assay using Vero cells was performed. Plaque formation was greater, which was indicated by fewer cells being stained with crystal violet, with the supernatants collected from MG1-infected cells compared with the supernatants from the birinapant-treated and MG1-infected cells (**Figure 3B**), suggesting that SMAC mimetics enhance the MG1-mediated killing via mechanisms other than increasing the MG1 infection.

### Upregulation of the immune response genes by the SMAC mimetics

**Figure 4:**
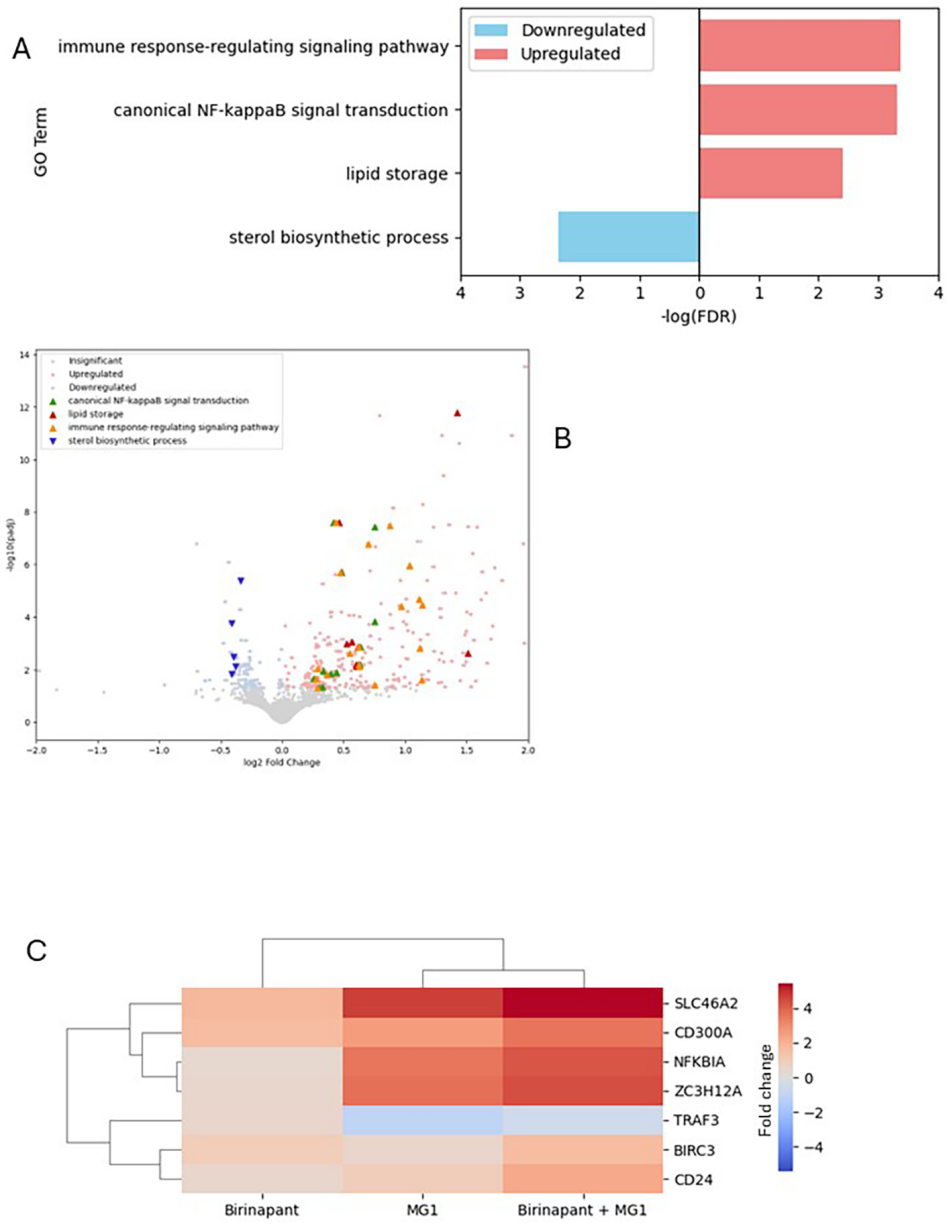
Immune response signaling is upregulated in J1.1 cells treated with MG1 and birinapant compared with cells only infected with MG1. The top 4 significantly enriched Gene Ontology (GO) terms (out of 27 terms in total) for *n* = 3 are shown in both. **(A)** GO biological process analysis. **(B)** Volcano plot. **(C)** Heat map showing the differentially expressed genes in the immune response signaling pathway.

To further investigate the changes induced by the SMAC mimetics at the gene level to sensitize cells to MG1-mediated death, RNA-seq was performed on J1.1 cells that were untreated, infected with MG1, or infected with MG1 and treated with SMAC mimetics. The top 4 GO terms in biological processes were plotted using differentially expressed genes (DEGs) via the g:Profiler. When cells treated with both the SMAC mimetics and MG1 were compared with cells only infected with MG1, upregulation in the genes that belong to the “immune response-regulating signaling pathway,” “canonical NF-κB transduction,” and “lipid storage” was observed. The only pathway in which a downregulation was observed was the “sterol biosynthesis pathway” (**Figures 4A, B**).

Following this, we investigated which genes were differentially expressed in the “immune response-regulating signaling pathway.” When comparing the cells that underwent combination treatment with the cells that were only infected with MG1, the following genes were found to be upregulated: *SLC46A2*, *CD300A*, *NFKBIA*, *ZC3H12A*, *BIRC3*, *TRAF3*, and *CD24* (**Figure 4C**). On the other hand, in the sterol biosynthesis pathway, the following genes were downregulated: *ERG28*, *DHCR24*, *LSS*, *SC5D*, and *LBR*.

### SMAC mimetics sensitize HIV-infected monocyte-derived macrophages to oncolytic virus-mediated death

MG1 has been shown to selectively kill HIV-infected MDMs (2). Therefore, we asked whether SMAC mimetics can increase the MG1-mediated killing of HIV-infected MDMs. As the rate of HIV infection is typically quite low in MDM cultures (26), we used an infection model previously optimized by our research group, where MDMs are infected with a replication-competent CCR5 tropic reporter virus, i.e., HIV NL4.3-BAL-IRES-HSA. Productively HIV-infected cells can be differentiated from bystander cells by the detection of mouse HSA on the cell surface using flow cytometry (2). At 6 days post-HIV infection, the MDMs were infected with MG1 and treated with the SMAC mimetic LCL-161 or birinapant. Cell death was measured 48 h later with annexin V staining in the bulk cell population and in the HSA-negative (HSA−) (bystander) and HSA-positive (HSA+) (HIV-infected) populations.

**Figure 5:**
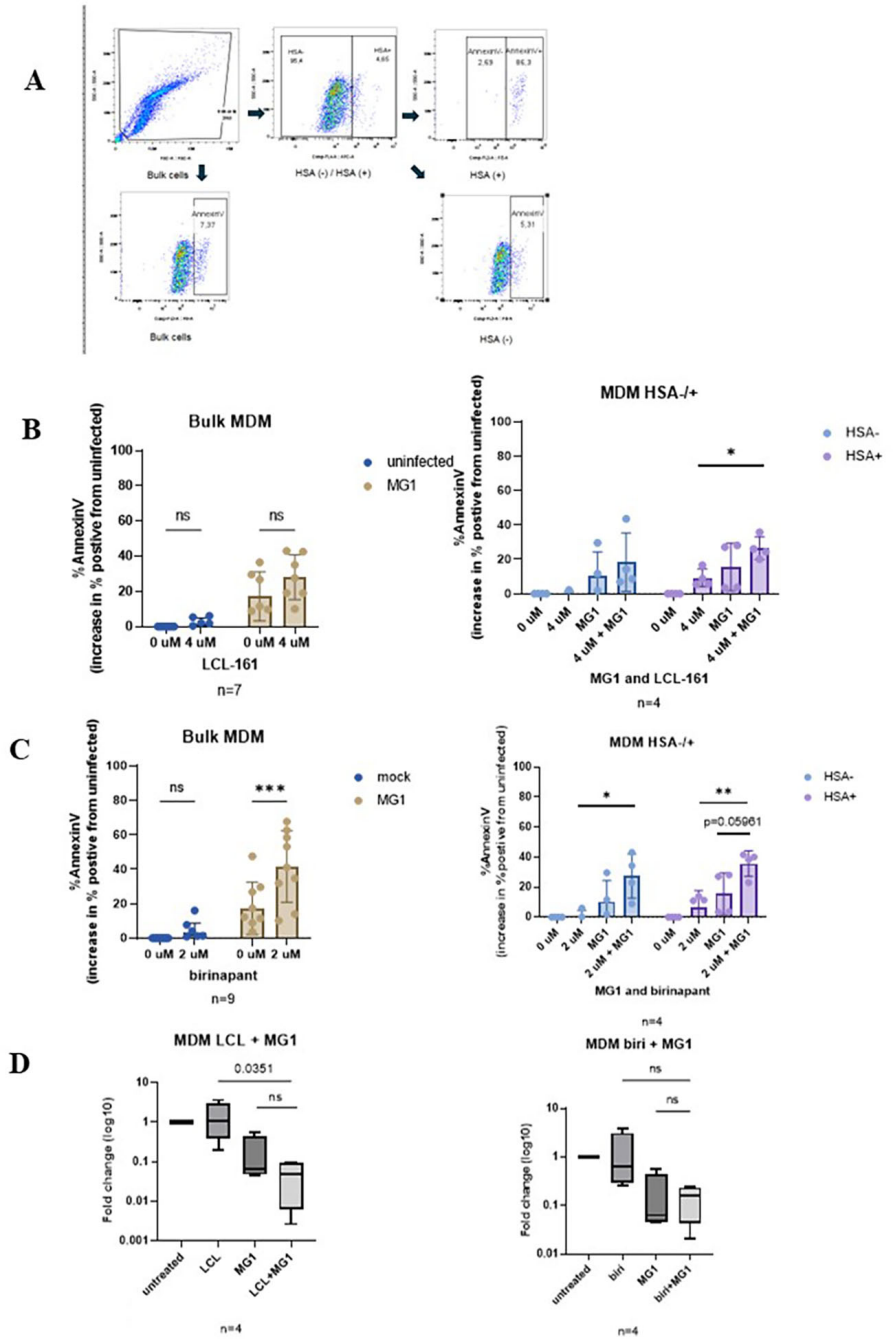
Treatment with the oncolytic virus MG1 with LCL-161 or birinapant kills HIV infected monocyte derived macrophages and decreases proviral HIV DNA. HIV infected MDM were infected with MG1 at MOI 1 and concurrently treated with the SMAC mimetics LCL-161 (4 µM) or birinapant (2 µM). Cell death was measured by AnnexinV staining and HIV infection status was determined by HSA staining via flow cytometry. Proviral HIV DNA was measured by qPCR. **(A)** Gating strategy employed for flow cytometry analysis **(B, C)** Cell viability of bulk MDM and HSA (-) and HSA (+) fractions following 48h concurrent treatment. 2-way analysis of variance with the Dunnet post-test for multiple comparisons between different treatment conditions. Data represent mean values ± standard deviation of the mean; n values represent separate biological replicates). **(D)** Proviral HIV DNA relative to untreated HIV infected cells. (n=4, p=0.0351 by Sidak’s post-test for MG1 and birinapant) Data represent mean ± SEM; n values represent separate biological replicates. *p<.05 **p<.01, ***p<.001. ns, not significant.

In agreement with our previous research, MG1 was shown to significantly kill MDMs in the bulk cell population (2). LCL-161 on its own did not induce the killing of MDMs, nor did it enhance MG1-mediated killing; however, the combination of LCL-161 and MG1 resulted in a significant increase in annexin-V-expressing cells compared with the untreated cells in the HSA+ (i.e., HIV-infected) population (**Figure 5B**). Moreover, while birinapant by itself did not cause cell death in the bulk cell population, the presence of birinapant significantly increased the MG1-mediated cell death in bulk MDMs (**Figure 5C**). Although there was a significant increase in annexin-V-expressing HSA+ cells that were treated with birinapant and MG1 in comparison to cells treated with birinapant alone, this increase in annexin V+ cells did not quite reach significance (*p* = 0.0596) (**Figure 5C**) when compared with the cells only treated with MG1. To further establish the decrease in the actual number of cells infected with HIV, quantitative PCR (qPCR) of the proviral HIV DNA was performed. Although there was a trend in decreasing proviral DNA in the MDMs treated with the SMAC mimetics and MG1, this difference did not reach statistical significance when compared with the cells only infected with MG1 (**Figure 5D**). These results indicate that, in our HIV infection model of MDMs, the SMAC mimetics did not consistently enhance MG1-mediated cell death. Considering the differences observed with LCL-161 and birinapant, it may be of interest to determine whether other SMAC mimetics may have the ability to synergize with MG1.

**Figure 6:**
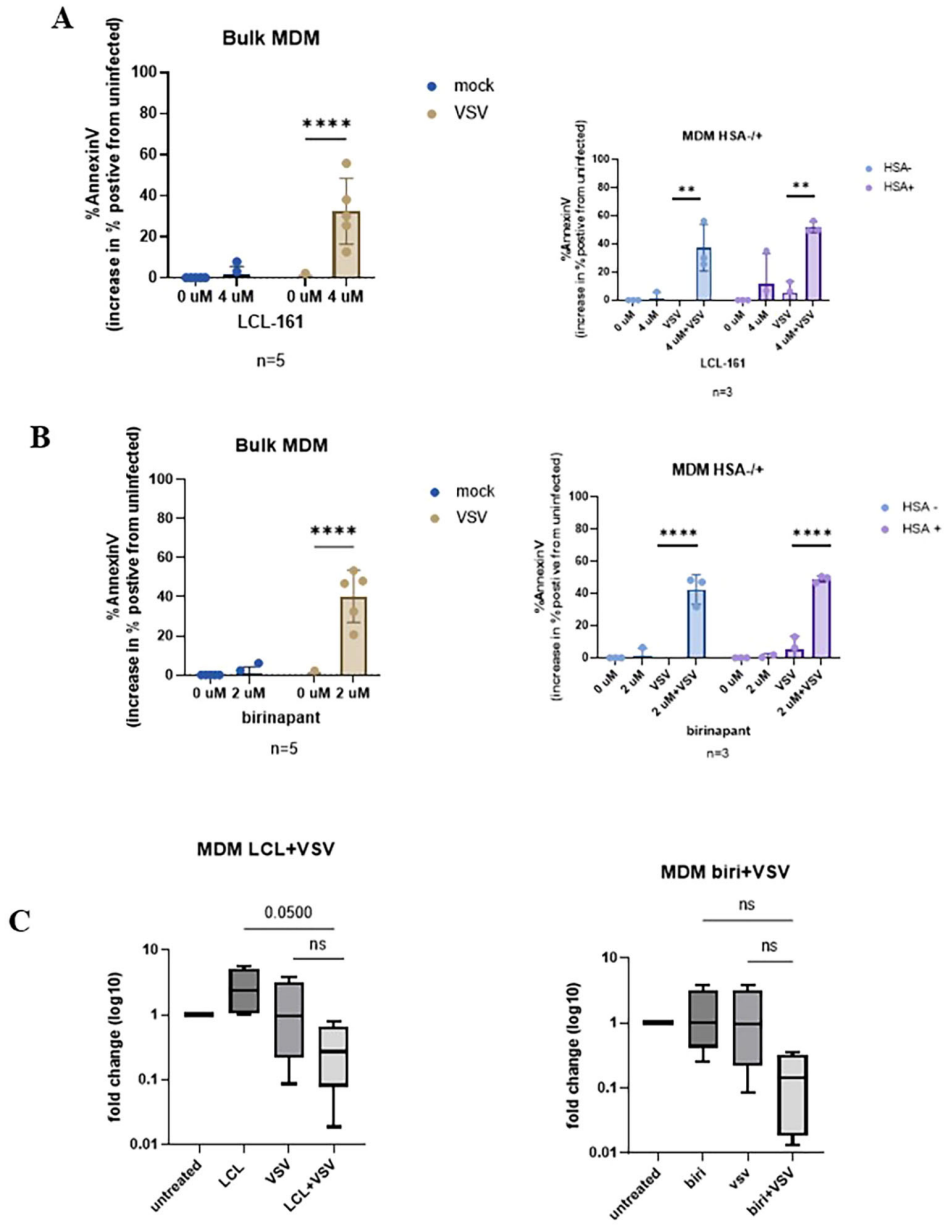
Treatment with the oncolytic virus VSVΔ51 with birinapant kills HIV-infected monocyte-derived macrophages (MDMs) and decreases the proviral HIV DNA. HIV-infected MDMs were infected with VSVΔ51 at a multiplicity of infection (MOI) of 1 and concurrently treated with the second mitochondria-derived activator of caspases (SMAC) mimetic birinapant (2 µM). Cell death was measured by annexin V staining and the HIV infection status determined by heat-stable antigen (HSA) staining via flow cytometry. Proviral HIV DNA was measured by quantitative PCR (qPCR). **(A, B)** Cell viability of the bulk MDM and HSA(−) and HSA(+) fractions following 48 h concurrent treatment. Two-way analysis of variance with the Dunnett post-test was used for multiple comparisons between the different SMAC mimetic doses. Data represent the mean value ± standard deviation of the mean. *n* values represent separate biological replicates). **(C)** Proviral HIV DNA relative to untreated HIV-infected cells (*n* = 4; two-way analysis of variance with Sidak’s post-test was performed). Data represent the mean ± SEM. *n* values represent separate biological replicates. **p<.01, ****p<.0001. ns, not significant.

Subsequently, we evaluated the ability of the SMAC mimetics to sensitize HIV-infected cells to death by another OV, namely, VSVΔ51. We have previously shown that VSVΔ51 alone is not capable of killing HIV-infected cell lines and HIV-infected MDMs (25, 27). OM10.1 cells were infected with VSVΔ51 and treated with the SMAC mimetic LCL-161 or birinapant. A significant decrease (*p* = 0.0039) in cell viability could be seen when the cells were infected with VSVΔ51 and treated with SMAC mimetics. Similarly to cells infected with MG1 and treated with SMAC mimetics, there was no change in the proportion of cells infected with this OV (**Supplementary Figure S4**). Thereafter, the HIV-infected MDMs were infected with VSVΔ51 and treated with SMAC mimetics. Interestingly, the addition of SMAC mimetics to VSVΔ51 significantly increased the cell death in both bulk cell populations and in the HSA+ and HSA− populations (**Figures 6A, B**). Similar to that observed with MG1, there appeared to be a decrease in proviral DNA, which did not reach statistical significance when compared with either treatment alone (**Figure 6C**).

### Secretion of TNFα in response to the oncolytic virus and SMAC mimetics

As previous studies have shown that the cell death of HIV-infected cells can be TNFα-dependent (10, 11) or TNFα-independent (12), we investigated the potential role of TNFα in OV infection- and SMAC mimetic-induced killing of HIV-infected MDMs. TNFα was measured using ELISA in the cell supernatants 48 h after OV (MG1 or VSVΔ51) infection, SMAC mimetic treatment, or OV plus SMAC mimetic treatment. While the OV infection resulted in detectable levels of TNFα in the cell supernatants, the SMAC mimetic treatment did not induce TNFα or enhance the OV-induced TNFα production (**Supplementary Figure S5**). Following this, we wanted to investigate whether the effect of OV infection on cell death can be replicated by treating the cells with saturating concentrations of exogenous TNFα. TNFα doses ranging between 5 and 20 ng/ml have been shown to not increase the SMAC mimetic-mediated killing of unpolarized (M0) or polarized (M1 and M2) macrophages (28). Similarly, in our model, no increase in cell death could be observed, implying that TNFα may not have a direct role in the cell death of HIV-infected MDMs (**Supplementary Figure S5**).

Subsequently, we investigated whether IFNα plays a role in SMAC mimetics sensitizing HIV-infected MDMs to OV-mediated death. IFNα was measured in the supernatants of HIV-infected MDMs, which were OV (MG1 or VSVΔ51)-infected, SMAC mimetic-treated, or OV- and SMAC mimetic-treated (**Supplementary Figure S5**) Interestingly, while no there was no detectable IFNα in the supernatants of the MDMs that were MG1-infected or MG1-infected and SMAC mimetic-treated, in conditions where the cells were treated with both VSVΔ51 and the SMAC mimetics, there was less IFNα present in the cell supernatants, suggesting that dampening the OV-induced IFN response is one method of SMAC mimetics sensitizing cells to VSVΔ51-mediated death.

## Discussion

Our research group has previously shown that the OV MG1 can selectively infect and kill latently HIV-infected CD4^+^ T cells (1) and persistently HIV-infected macrophages (2). In the present study, we demonstrated that pro-apoptotic SMAC mimetics can enhance the OV-mediated killing of HIV-infected cells.

Several studies have shown that HIV infection results in the upregulation of anti-apoptotic proteins. For example, CD4^+^ T cells that have been transfected with HIV Tat have been shown to upregulate the expression of the anti-apoptotic cFLIP and Bcl-2 (16, 18). Thus, the use of anti-apoptotic small-molecule SMAC mimetics to kill HIV-infected cells is a popular area of research. SMAC mimetics have been shown to kill latently HIV-infected cell lines (9, 11), latently HIV-infected CD4^+^ T cells (29), and HIV-infected macrophages (10). Furthermore, SMAC mimetics are being used to enhance OV-mediated killing in several different cancers (3–5). These studies have emphasized the importance of the order of administration of OVs and SMAC mimetics, as in one model where EMT6 tumor-bearing mice were used, the administration of SMAC mimetics prior to OV infection resulted in the loss of OV infection and the prevention of cancer cell killing (5).

Here, we showed that the bivalent SMAC mimetics AEG-40730 and birinapant and the monovalent SMAC mimetic LCL-161 increased cell death in latently HIV-infected cell lines and in HIV-infected MDMs mediated by the OVs MG1 and VSVΔ51. This effect was observed in the HIV-infected daughter cell line J1.1, but not in the parental Jurkat cell line, suggesting a degree of selectivity for HIV infection. In addition, we have shown that only pretreatment with AEG-40730 was able to increase the MG1-mediated killing in OM10.1 cells, reinforcing the concept that the order of treatment is indeed important.

SMAC mimetics have been shown to kill HIV-infected cells via a combination of apoptosis and autophagy in different infection models (10, 29). On the other hand, studies on how OVs, specifically MG1, kill HIV-infected cells are limited. Preliminary work by our group has shown that the caspase inhibitors Z-VAD-FMK and Z-DEVD-FMK are able to somewhat mitigate the MG1-mediated killing of HIV-uninfected cell lines, but these caspase inhibitors were unable to significantly block cell death in latently HIV-infected cells (30). Furthermore, both the pan-caspase inhibitor Z-VAD-FMK and the necroptosis inhibitor necrostatin-1s failed to inhibit the cell death of HIV-infected macrophages (27). Thus, we investigated the cell death pathways responsible for SMAC mimetic- and MG1-mediated killing. We have shown here that there is increased activation of both caspase-3/7 and caspase-1 in J1.1 cells, indicating that the HIV-infected cells may be undergoing both apoptosis and pyroptosis. Furthermore, when we used the necroptosis inhibitor necrostatin-1s along with Z-VAD-FMK to block cell death, we observed that cell death was completely blocked in cells treated with the SMAC mimetics alone, but with MG1-mediated cell death persisting, suggesting that MG1 induces cell death via mechanisms in addition to those explored above. Recent studies have shown that cell death caused by VSV infection can only be blocked by inhibiting all three of the apoptosis, necroptosis, and pyroptosis pathways and have coined the term PANoptosis for this type of cell death (31). Considering the close genetic relationship between VSV and MG1 (32), we hypothesize that MG1 might cause cell death via a similar mechanism.

In attempts to determine the mechanism(s) by which SMAC mimetics sensitize HIV-infected cells to MG1-mediated death, a number of experiments were performed, which demonstrated that the SMAC mimetics neither increased infection with MG1 nor increased MG1 replication in these cells. To further address potential pathways by which SMAC mimetics mediated this activity, RNA-seq experiments were performed.

The GO analysis for biological processes showed that the DEGs clustered in upregulation of the “immune response signaling pathway,” “canonical NFκB transduction,” and “lipid storage” and in downregulation of the “sterol biosynthesis pathway” when J1.1 cells were concurrently treated with MG1 and birinapant. The genes that were shown to be downregulated were *ERG28*, *DHCR24*, *LSS*, *SC5D*, and *LBR* in the sterol biosynthesis pathway, while the upregulated genes were *CD24*, *SLC46A2*, *ZC3H12A*, *TRAF3*, *NFKBIA*, and *BIRC3* in the immune response signaling pathway.

HIV infection has been shown to be involved in the regulation of the genes responsible for the cholesterol biosynthesis pathway in different HIV-infected T-cell lines (33). Moreover, murine cytomegalovirus (mCMV) models have shown that the production of IFNβ and IFNγ in response to viral infection results in the transcriptional downregulation of the sterol biosynthesis pathway in bone marrow-derived macrophages (34). These studies suggest that the downregulation of the sterol biosynthesis pathway may be a response to viral infection.

While the role of CD24 in T cells is mainly in proliferation (35), it has also been implicated to play a role in cell death in B cells (36), neutrophils (37), and thymocytes (38). Moreover, in a neuroblastoma infection model of Zika virus, CD24 has been shown to dampen the antiviral response by decreasing the response to IFN1 and thus increasing permissivity to RNA virus infection (39, 40).

*SLC46A2* encodes for a cGAMP transporter in response to double-stranded DNA (41), which then exerts antiviral effects. *ZC3H12A* encodes the protein called MCPIP-1 (also called Regnase-1) (42). MCPIP-1 is typically induced following exposure to inflammatory cytokines (43, 44), stimulation by MCP-1 (45), or infection (46). Its main role is to aid in the resolution of the inflammatory response to return to homeostasis by modulating the inflammatory cytokines at both the transcriptional and post-transcriptional levels (47). MCPIP-1 is also upregulated in response to viral infection, as it has the ability to restrict viral replication by causing the selective degradation of viral RNAs (48, 49).

*TRAF3*, *NFKBIA*, and *BIRC3* are genes that all participate in the NF-κB signaling pathway for the activation of pro-inflammatory genes, which results in antiviral immunity (50). These results agree with other studies that have shown the upregulation of *BIRC3* as a response to the degradation of cIAP2 by birinapant (14).

Although these upregulated genes appear to have functions ranging from increasing the permissiveness of RNA viruses to antiviral immunity, further research is required to validate the expression of these genes at the protein level in order to fully elucidate the changes that occur following the addition of SMAC mimetics to MG1 infection.

Subsequently, using an established model involving HIV infection of primary MDMs (2, 26), we chose to move forward with LCL-161 and birinapant to determine whether they sensitize cells to MG1-mediated cell death, as both of these SMAC mimetics are currently in clinical trials for cancer (NCT02098161 and NCT04553692, respectively). Here, we have shown that, although there was an increase in cell death in the HSA+ population compared with the HSA− population following concurrent MG1 infection and SMAC mimetic treatment, this increase was not significant. A similar observation was made when the decrease in proviral HIV DNA was used as a measure of cell killing. Interestingly, although other studies have shown that SMAC mimetics selectively kill HIV-infected MDMs (10, 11), neither LCL-161 nor birinapant treatment of MDMs resulted in significant cell death in either bulk or individual HSA−/HSA+ populations in our infection model, emphasizing the importance of the HIV infection models used.

We next showed that, while VSVΔ51 itself did not kill HIV-infected MDMs, the addition of SMAC mimetics to these cells resulted in a significant increase in VSVΔ51-mediated cell death, as demonstrated by both flow cytometric measures of cell death and the decrease in proviral HIV DNA. However, under these experimental conditions, there did not appear to be selectivity for HIV-infected cells. This indicates that, although VSVΔ51 and MG1 are very similar genetically (32), their mechanisms of action when killing HIV-infected cells may differ.

As SMAC mimetic treatment can cause TNFα-dependent and TNFα-independent cell death of HIV-infected MDMs (10, 11), we investigated the potential role of TNFα in HIV-infected MDMs following treatment with the OV and SMAC mimetics. Interestingly, while the SMAC mimetic treatment alone did not result in any detectable production of TNFα, there were significant amounts of TNFα measured in the supernatants of the HIV-infected MDMs that were infected with OV or were infected with OV and treated with SMAC mimetics. This finding is consistent with previous literature on MG1 and VSVΔ51 infection, where it has been shown that infection with these OVs results in the release of pro-inflammatory cytokines such as TNFα, IL-4, and IL-6 (2, 51, 52). However, when the MDMs were pre-incubated with TNFα and then treated with SMAC mimetics, there was no significant increase in cell death. Previous studies have shown that treatment with exogenous TNFα and LCL-161 significantly increases cell death compared with either treatment alone in HIV-infected cell lines, but not in HIV-infected MDMs (11), which is consistent with our results.

Previous work from our group has shown that IFNα treatment protects HIV-infected MDMs from MG1 infection (2). Hence, we further investigated the role of IFNα in the context of OV infection and SMAC mimetic treatment. Infection of HIV-infected MDMs with MG1 or their treatment with SMAC mimetics did not result in any detectable IFNα in the supernatants. However, infection with VSVΔ51 and treatment with SMAC mimetics resulted in a non-significant but detectable decrease in IFNα.

Although the SMAC mimetics and OVs by themselves have been shown to be unable to induce significant killing of CD4^+^ T cells, this combination strategy has never been examined on HIV-infected or uninfected CD4^+^ T cells. To move this possible treatment option to the clinic, it is important for this combination treatment to be tested in this cell population in order to ensure that the treatment does not affect the CD4^+^ T-cell counts of individuals living with HIV.

In summary, we have demonstrated that the bivalent SMAC mimetics AEG-40730 and birinapant and the monovalent SMAC mimetic LCL-161 sensitize HIV-infected cell lines and HIV-infected MDMs to MG1-mediated cell death via caspase-dependent and caspase-independent pathways. Moreover, we have shown that SMAC mimetics can also be used to sensitize HIV-infected cell lines and MDMs to killing using VSVΔ51. To our knowledge, this is the first study to employ combination therapy with OV and SMAC mimetics to eliminate latently HIV-infected cells. These findings indicate that the novel strategy using SMAC mimetics and OV infection might synergize to specifically kill HIV-infected cells and may be a useful tool in our arsenal to fight HIV infection.

## Data Availability

The names of the repository/repositories and accession number(s) can be found in the article/Materials and Methods - RNA-seq analysis.
