# Supplementary material for: SMAC mimetics sensitize HIV-infected cells to oncolytic virus-mediated death
Source: Front Immunol. 2025 Dec 18;16:1665811. doi: 10.3389/fimmu.2025.1665811 (PMC12756378; doi:10.3389/fimmu.2025.1665811)
Supplement: Supplementary file 1 [file Presentation1.pptx]

## Slide 1
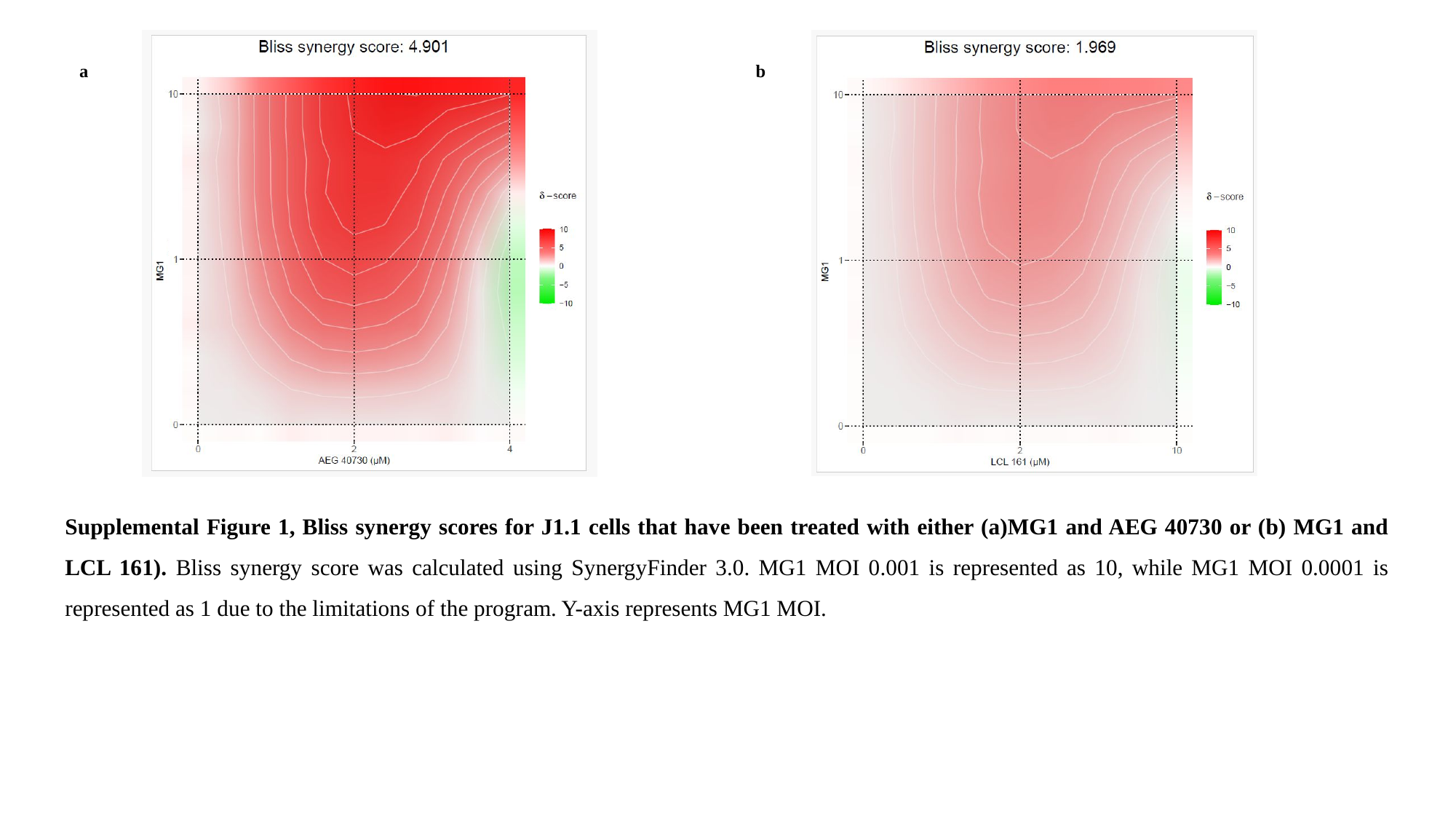

a
b
Supplemental Figure 1, Bliss synergy scores for J1.1 cells that have been treated with either (a)MG1 and AEG 40730 or (b) MG1 and LCL 161). Bliss synergy score was calculated using SynergyFinder 3.0. MG1 MOI 0.001 is represented as 10, while MG1 MOI 0.0001 is represented as 1 due to the limitations of the program. Y-axis represents MG1 MOI.

## Slide 2
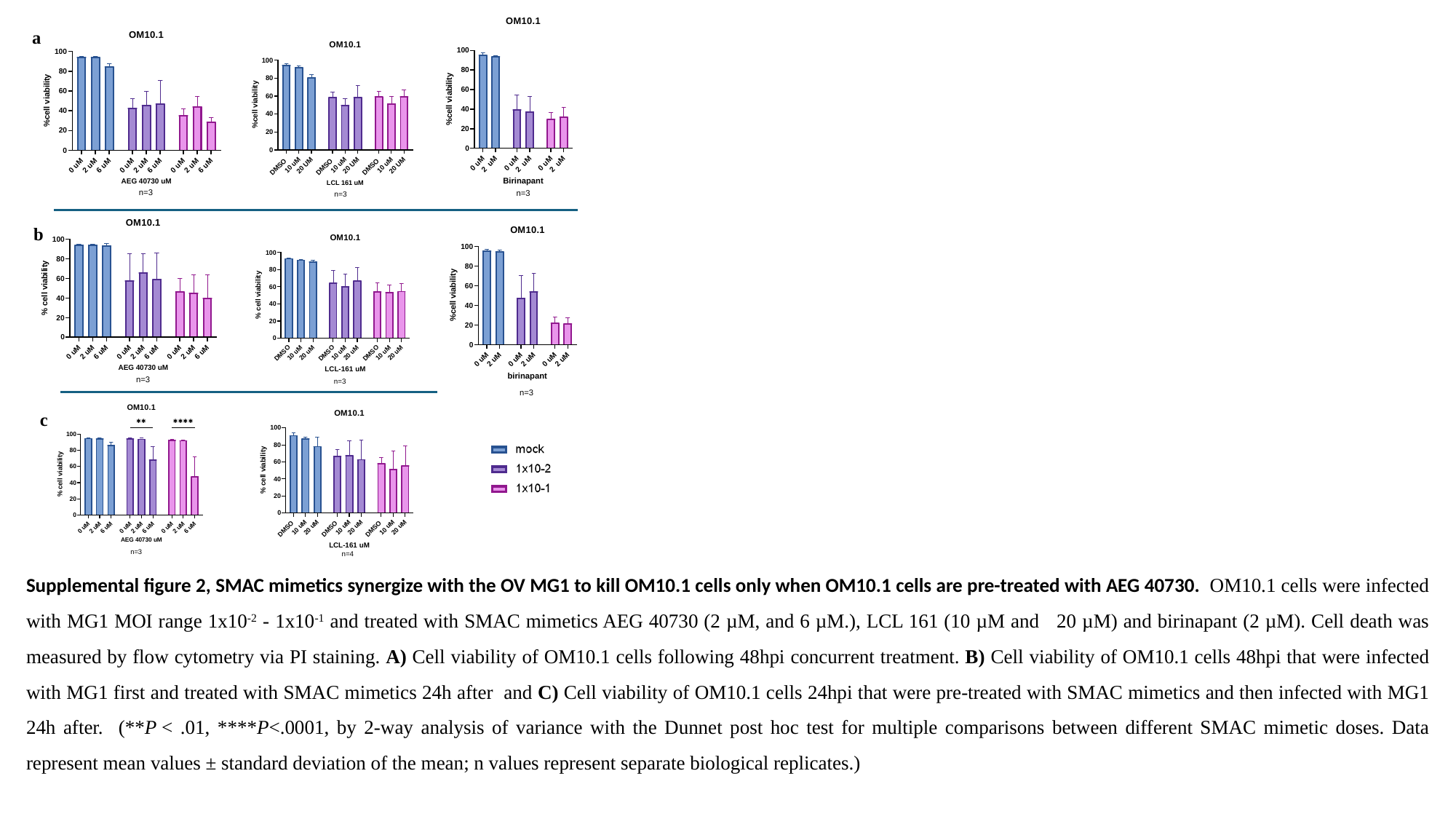

a
b
c
Supplemental figure 2, SMAC mimetics synergize with the OV MG1 to kill OM10.1 cells only when OM10.1 cells are pre-treated with AEG 40730. OM10.1 cells were infected with MG1 MOI range 1x10^-2^ - 1x10^-1^ and treated with SMAC mimetics AEG 40730 (2 µM, and 6 µM.), LCL 161 (10 µM and 20 µM) and birinapant (2 µM). Cell death was measured by flow cytometry via PI staining. A) Cell viability of OM10.1 cells following 48hpi concurrent treatment. B) Cell viability of OM10.1 cells 48hpi that were infected with MG1 first and treated with SMAC mimetics 24h after and C) Cell viability of OM10.1 cells 24hpi that were pre-treated with SMAC mimetics and then infected with MG1 24h after. (**P < .01, ****P<.0001, by 2-way analysis of variance with the Dunnet post hoc test for multiple comparisons between different SMAC mimetic doses. Data represent mean values ± standard deviation of the mean; n values represent separate biological replicates.)

## Slide 3
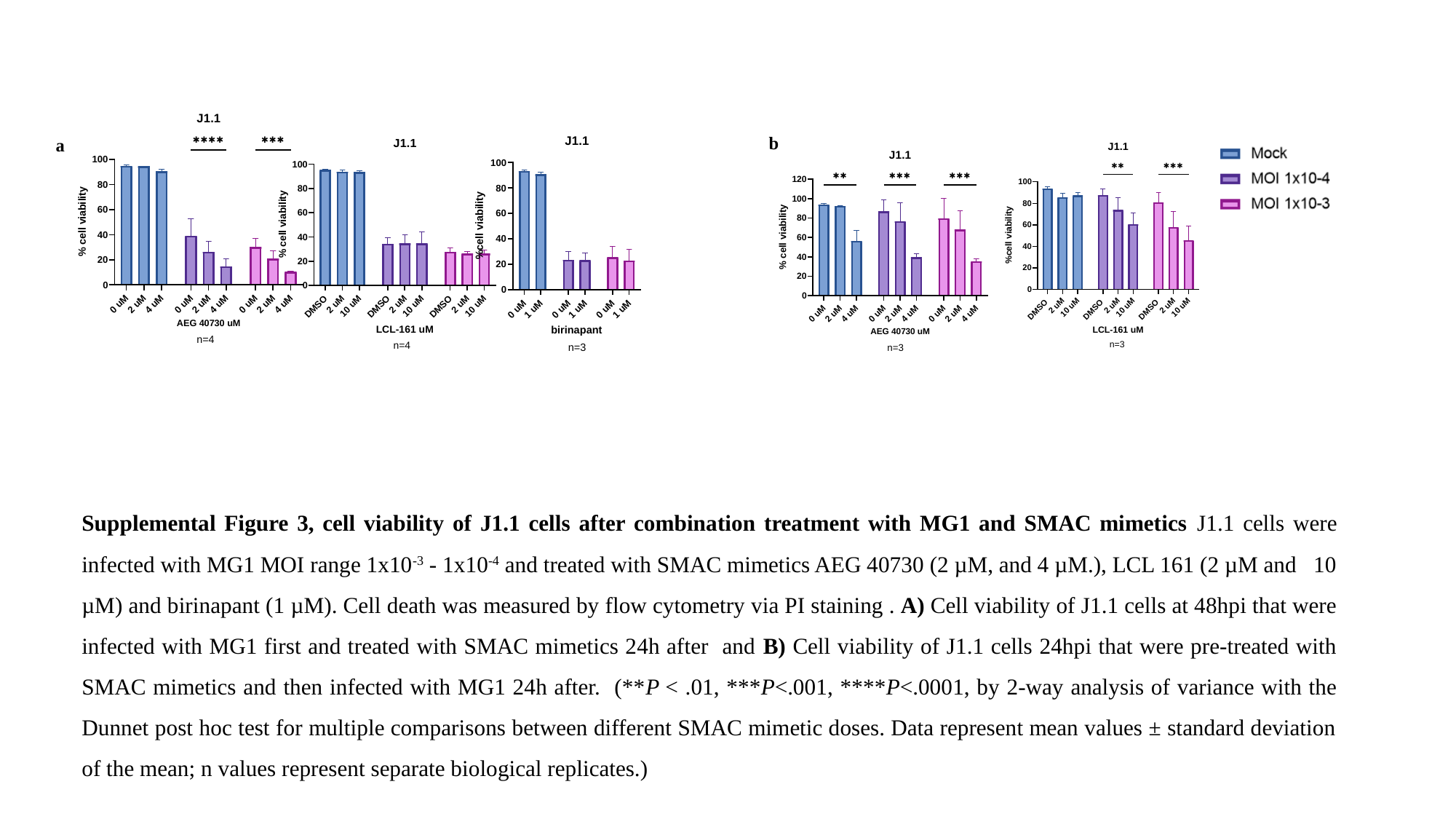

b
a
Supplemental Figure 3, cell viability of J1.1 cells after combination treatment with MG1 and SMAC mimetics J1.1 cells were infected with MG1 MOI range 1x10^-3^ - 1x10^-4^ and treated with SMAC mimetics AEG 40730 (2 µM, and 4 µM.), LCL 161 (2 µM and 10 µM) and birinapant (1 µM). Cell death was measured by flow cytometry via PI staining . A) Cell viability of J1.1 cells at 48hpi that were infected with MG1 first and treated with SMAC mimetics 24h after and B) Cell viability of J1.1 cells 24hpi that were pre-treated with SMAC mimetics and then infected with MG1 24h after. (**P < .01, ***P<.001, ****P<.0001, by 2-way analysis of variance with the Dunnet post hoc test for multiple comparisons between different SMAC mimetic doses. Data represent mean values ± standard deviation of the mean; n values represent separate biological replicates.)

## Slide 4
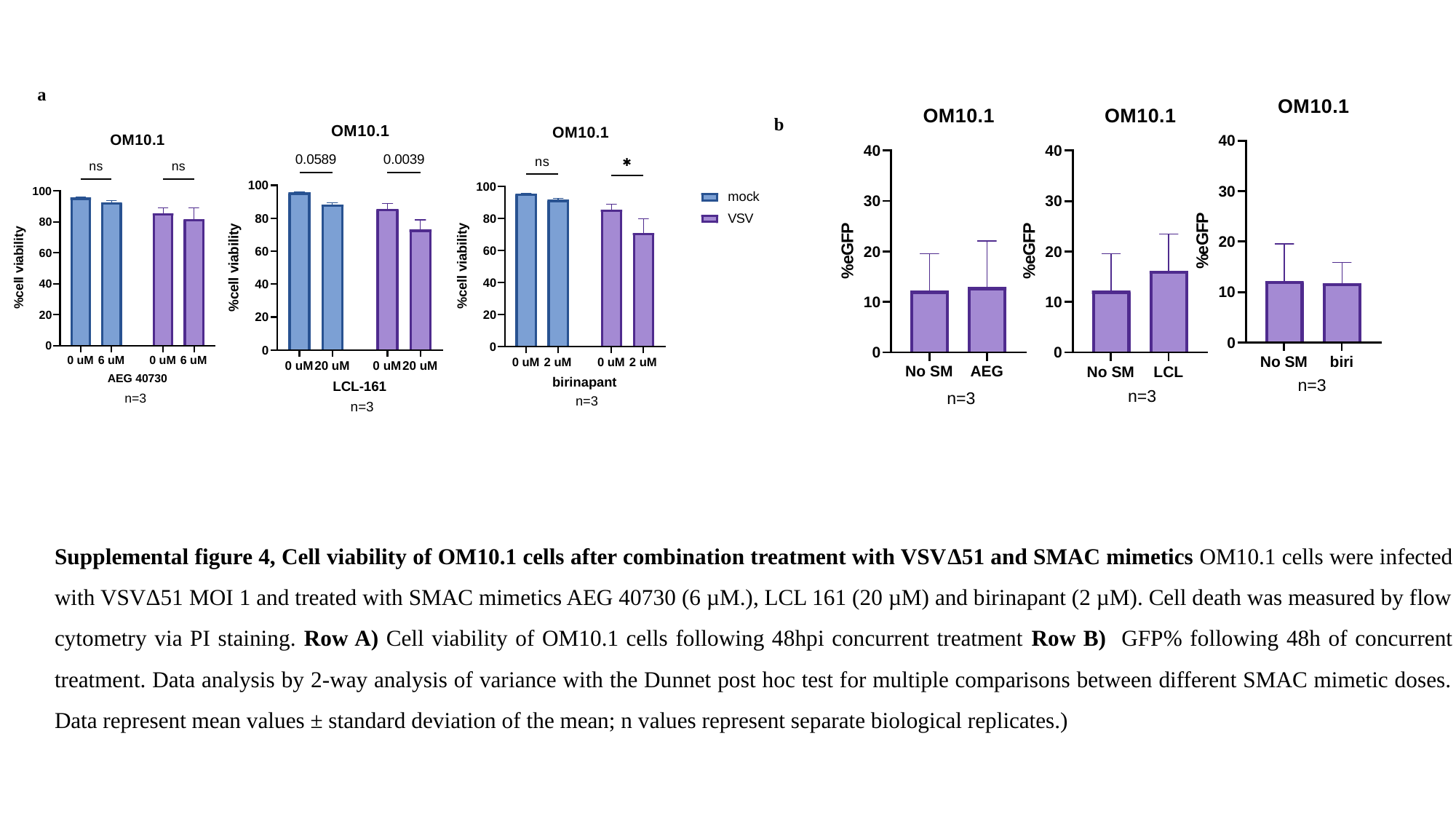

a
b
Supplemental figure 4, Cell viability of OM10.1 cells after combination treatment with VSVΔ51 and SMAC mimetics OM10.1 cells were infected with VSVΔ51 MOI 1 and treated with SMAC mimetics AEG 40730 (6 µM.), LCL 161 (20 µM) and birinapant (2 µM). Cell death was measured by flow cytometry via PI staining. Row A) Cell viability of OM10.1 cells following 48hpi concurrent treatment Row B) GFP% following 48h of concurrent treatment. Data analysis by 2-way analysis of variance with the Dunnet post hoc test for multiple comparisons between different SMAC mimetic doses. Data represent mean values ± standard deviation of the mean; n values represent separate biological replicates.)

## Slide 5
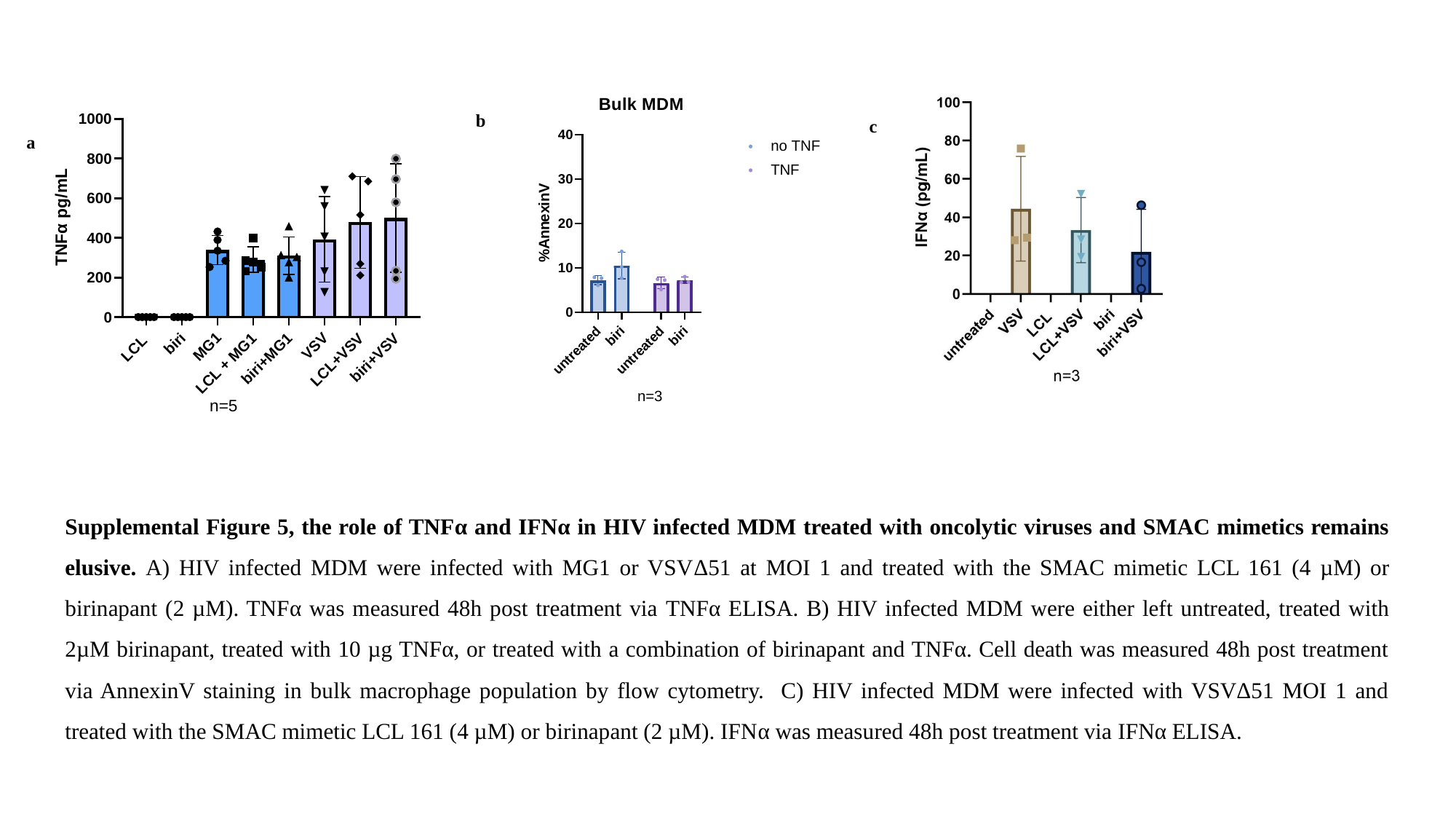

b
c
a
Supplemental Figure 5, the role of TNFα and IFNα in HIV infected MDM treated with oncolytic viruses and SMAC mimetics remains elusive. A) HIV infected MDM were infected with MG1 or VSVΔ51 at MOI 1 and treated with the SMAC mimetic LCL 161 (4 µM) or birinapant (2 µM). TNFα was measured 48h post treatment via TNFα ELISA. B) HIV infected MDM were either left untreated, treated with 2µM birinapant, treated with 10 µg TNFα, or treated with a combination of birinapant and TNFα. Cell death was measured 48h post treatment via AnnexinV staining in bulk macrophage population by flow cytometry. C) HIV infected MDM were infected with VSVΔ51 MOI 1 and treated with the SMAC mimetic LCL 161 (4 µM) or birinapant (2 µM). IFNα was measured 48h post treatment via IFNα ELISA.
